# Asymptomatic hair shaft nodules in pediatric siblings: A diagnostic challenge

**DOI:** 10.1016/j.jdcr.2026.03.050

**Published:** 2026-03-30

**Authors:** Raed A.H. Shabaa, Rand M.A. Al-Husseini, Ayat Q. Abdul Wahid, Fatima A. Nayef, Saja H. Abdul Razzaq, Fatima K. Aboud

**Affiliations:** a Department of Pathological Analyses, Faculty of Science, University of Kufa, Najaf, Iraq; b Department of Biology, Faculty of Science, University of Kufa, Najaf, Iraq

**Keywords:** hair shaft disorder, hair shaft nodules, mycology, pediatric dermatology, superficial fungal infection

## Case description

**Fig 1:**
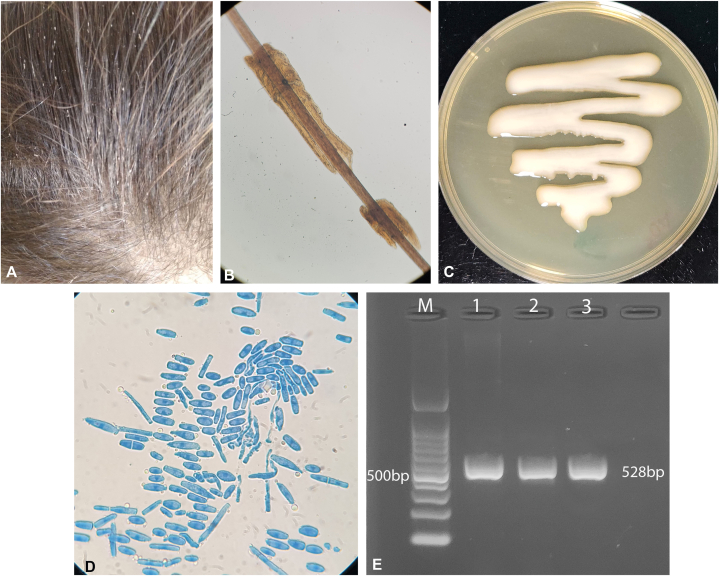
Pediatric white piedra caused by *Cutaneotrichosporon mucoides*. **A,** Whitish nodules firmly adherent along scalp hair shafts. **B,** Light microscopy showing nodular fungal aggregates encasing the hair shaft (×100). **C,** Creamy, mucoid colony morphology on Sabouraud dextrose agar. **D,** Microscopy of cultured isolate showing septate hyphae with blastoconidia and arthroconidia. **E,** Agarose gel electrophoresis showing a single ∼528 bp ITS1-5.8S-ITS2 amplicon (lanes 1-3); M, DNA marker.

Three pediatric female siblings aged 4, 6, and 12 years presented with asymptomatic hair shaft abnormalities. Physical examination revealed multiple soft, whitish nodules firmly adherent to scalp hair shafts, producing a speckled to beaded appearance (Fig 1, *A*). No surrounding scalp inflammation was observed.

Affected hair strands were clipped close to the scalp using sterile scissors. Direct light microscopy demonstrated compact nodular fungal aggregates encasing the external surface of the hair shafts without Penetration into the cortex, consistent with a superficial hair shaft infection (Fig 1, *B*). Hair samples were cultured on Sabouraud dextrose agar and incubated at 28-30 °C. Cultures yielded creamy, moist, mucoid yeast-like colonies (Fig 1, *C*).

Microscopic examination of cultured isolates revealed hyaline septate hyphae with abundant oval blastoconidia and rectangular to barrel-shaped arthroconidia (Fig 1, *D*), suggesting a yeast-like basidiomycetous fungus. Amplification of the internal transcribed spacer (ITS) region produced a single ∼528-bp amplicon on agarose gel electrophoresis (Fig 1, *E*), and sequencing confirmed *Cutaneotrichosporon mucoides*.

**Quiz Question: What is the most likely diagnosis?**

**A.** *Pediculosis capitis*
**B.** *Trichoblastosis nodularis*
**C.** Black piedra (*Piedraia hortae*)
**D.** White piedra caused by *Cutaneotrichosporon mucoides*
**E.** White piedra caused by *Trichosporon asahii*

Correct answer: **D.**

## Discussion

White piedra is an uncommon superficial fungal infection of the hair shaft characterized by soft, whitish nodules firmly adherent to hair fibers and resistant to routine washing, without associated scalp inflammation.^1^^,^^2^ The clinical presentation in this case, including asymptomatic hair shaft nodules and absence of scalp erythema or alopecia, is characteristic of white piedra (Fig 1, *A* and *B*).

*Pediculosis capitis* (Answer A) typically presents with pruritus and visible nits attached near the scalp surface rather than discrete fungal nodules encasing the hair shaft. *Trichorrhexis nodosa* (Answer B) represents a structural hair shaft defect causing fraying and breakage rather than true nodular concretions. Black piedra caused by *P. hortae* (Answer C) produces hard, dark, firmly attached nodules that differ clinically from the soft, light-colored concretions observed in this case. White piedra caused by *T. asahii* (Answer E) shares similar clinical features; however, species differentiation requires microbiologic or molecular identification.

Direct microscopy typically demonstrates fungal aggregates encasing hair shaft surface without cortical invasion, helping to distinguish white piedra from dermatophyte infections, which usually show endothrix or ectothrix invasion,^1^ while culture revealed creamy, mucoid colonies consistent with *Cutaneotrichosporon* species^3^ (Fig 1, *C* and *D*). Molecular sequencing confirmed identification as *C. mucoides*, a rare etiologic agent of pediatric white piedra.

Because yeast-like fungi show phenotypic overlap, morphologic identification alone is insufficient for definitive species assignment. Molecular identification is the reference standard for species-level identification of medically important fungi and was essential for confirming *C. mucoides* in this case.^4^ Classical culture- and microscopy-based methods remain fundamental for diagnosing superficial fungal infections, as demonstrated in previous clinical mycology studies.^5^

The occurrence of white piedra in siblings suggests shared environmental exposure or similar hair-care practices rather than host immunodeficiency. This case highlights the importance of integrating clinical evaluation, conventional mycology, and molecular methods for accurate diagnosis of hair shaft mycoses.

## Conflicts of interest

None disclosed.
